# Supplementary material for: Age-Dependent Composition and Diversity of the Gut Microbiome in Endangered Gibbon (Nomascus hainanus) Based on 16S rDNA Sequencing Analysis
Source: Microorganisms. 2025 May 26;13(6):1214. doi: 10.3390/microorganisms13061214 (PMC12195303; doi:10.3390/microorganisms13061214)
Supplement: Supplementary file 1 [file microorganisms-13-01214-s001.zip › Supplementary Table S1. Sequencing data and quality statistics..pdf]

Supplementary Table S1. Sequencing data and quality statistics

Sample Name	Raw Tags	Clean Tags	Effective Tags	Effective Ratio (%)	Max length	Min length	N50	OTUs
J1	87551	87092	70625	79.21	477	206	461	285
J2	89551	89088	79638	87.07	478	203	461	270
J3	125651	125083	97559	75.72	472	208	461	955
J4	130760	130176	101683	75.85	474	206	461	1023
J5	70873	70410	65315	90.47	478	223	461	214
J6	98781	98375	78519	78.07	477	213	461	297
J7	85335	84979	71274	81.97	478	217	461	295
S1	119813	118838	94669	77.75	478	202	461	346
S2	124961	124087	99729	78.25	478	202	461	402
S3	118668	117411	92788	76.89	478	201	461	357
S4	125480	124123	96897	76.05	478	201	461	437
S5	123963	122967	96484	76.56	478	202	461	415
S6	119087	118252	92248	76.4	478	201	461	417
S7	125995	124864	98893	77.23	478	202	461	459
S8	122541	121741	97376	78.14	478	201	461	384
S9	122931	122129	99365	79.65	478	201	461	352
S10	127863	126858	99449	76.53	477	202	461	435
S11	129249	128486	103757	79.05	478	201	461	398
S12	119996	119160	95912	78.63	478	202	461	365
S13	126640	125322	97536	75.86	478	202	461	419
S14	134696	133759	105751	77.24	478	202	461	405
S15	122845	121844	95654	76.69	478	202	461	387
E1	125335	124372	98315	77.06	478	202	461	403
E2	118293	117470	91416	76.13	478	201	461	394
E3	134514	133483	105562	77.1	478	202	461	430
E4	124670	123840	97740	77.05	478	201	461	397
E5	126966	126085	99928	77.38	478	201	461	403
E6	128722	127499	99857	76.37	478	202	461	435
E7	123989	123067	98647	78.14	478	201	461	391
E8	130072	129130	101728	77.01	478	201	461	407
E9	119159	118135	93366	77.12	478	202	461	403
E10	134742	133981	105545	77.15	478	206	461	414
E11	120972	120133	96151	78.14	478	202	461	362
E12	126230	124826	98304	76.56	478	201	461	411
E13	135420	134439	107350	78.01	478	201	461	381
E14	125765	124840	99060	77.5	478	201	461	401
E15	118938	118260	98418	81.37	477	204	461	346
E16	118910	118059	93261	77.25	478	201	461	367
E17	134055	133042	108651	79.58	478	202	461	409

E18	135659	134182	106822	77.51	478	201	461	427
E19	133953	132832	104913	77.23	478	201	461	416
