# Supplementary figures and images for: Age-Dependent Composition and Diversity of the Gut Microbiome in Endangered Gibbon (Nomascus hainanus) Based on 16S rDNA Sequencing Analysis
Source: Microorganisms. 2025 May 26;13(6):1214. doi: 10.3390/microorganisms13061214 (PMC12195303; doi:10.3390/microorganisms13061214)

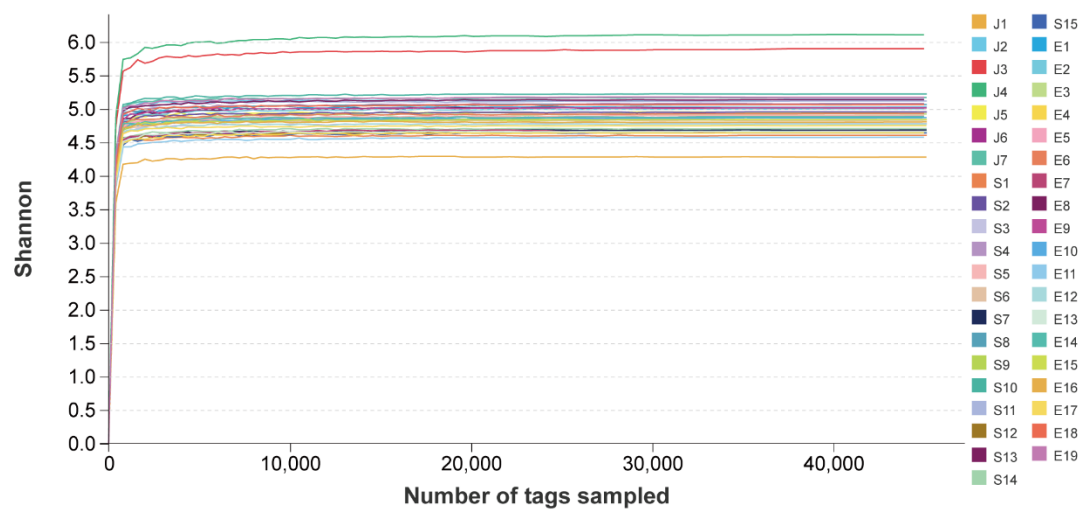

**Supplementary Figure 1.** Shannon diversity rarefaction curves.

Supplement: Supplementary file 1 [file microorganisms-13-01214-s001.zip › Supplementary Figure 1. Shannon diversity rarefaction curves..pdf]
